# Many Activities, One Structure: Functional Plasticity of Ribozyme Folds

**DOI:** 10.3390/molecules21111570

**Published:** 2016-11-18

**Authors:** Matthew W.L. Lau, Adrian R. Ferré-D’Amaré

**Affiliations:** National Heart, Lung and Blood Institute, 50 South Drive, MSC 8012, Bethesda, MD 20892-8012, USA; matt.wl.lau@gmail.com

**Keywords:** ribozyme, in vitro selection, fitness landscape

## Abstract

Catalytic RNAs, or ribozymes, are involved in a number of essential biological processes, such as replication of RNA genomes and mobile genetic elements, RNA splicing, translation, and RNA degradation. The function of ribozymes requires the formation of active sites decorated with RNA functional groups within defined three-dimensional (3D) structures. The genotype (sequence) of RNAs ultimately determines what 3D structures they adopt (as a function of their environmental conditions). These 3D structures, in turn, give rise to biochemical activity, which can further elaborate them by catalytic rearrangements or association with other molecules. The fitness landscape of a non-periodic linear polymer, such as RNA, relates its primary structure to a phenotype. Two major challenges in the analysis of ribozymes is to map all possible genotypes to their corresponding catalytic activity (that is, to determine their fitness landscape experimentally), and to understand whether their genotypes and three-dimensional structures can support multiple different catalytic functions. Recently, the combined results of experiments that employ in vitro evolution methods, high-throughput sequencing and crystallographic structure determination have hinted at answers to these two questions: while the fitness landscape of ribozymes is rugged, meaning that their catalytic activity cannot be optimized by a smooth trajectory in sequence space, once an RNA achieves a stable three-dimensional fold, it can be endowed with distinctly different biochemical activities through small changes in genotype. This functional plasticity of highly structured RNAs may be particularly advantageous for the adaptation of organisms to drastic changes in selective pressure, or for the development of new biotechnological tools.

## 1. Ribozyme Function and Evolution

RNAs are involved in a range of cellular processes, and an important capability that was not discovered until the 1980s is their ability to function as catalysts [1,2]. RNA enzymes, or ribozymes, can mediate a broad range of chemistries using a limited range of functional groups. In nature, ribozymes are present in all three kingdoms, and some can achieve catalytic efficiencies comparable to those of protein enzymes [3]. Surprisingly few classes of natural ribozymes have been discovered to date. The most sophisticated example is the ribosome [4], which catalyzes the messenger RNA (mRNA)-directed peptidyl transfer reaction that is essential in all living organisms. The self-splicing introns and ribonuclease P (RNase P) are phylogenetically widespread ribozymes that are involved in pre-mRNA and pre-transfer RNA (tRNA) processing, respectively, and catalyze phosphoryl transfer reactions. Self-splicing introns are often components of mobile genetic elements and play key roles in their mobilization. Most of the known classes of natural ribozymes are classified as “small self-cleaving ribozymes”. The hairpin, hammerhead, hatchet, hepatitis delta virus, *glmS*, pistol, twister, twister sister, and Varkud satellite ribozymes all catalyze internal phosphoryl transfer in processes such as RNA genome replication and the regulation of gene expression at the mRNA level [5,6]. In addition to these, many artificial ribozymes, which catalyze a much broader range of chemical reactions, have been discovered through in vitro selection methods [7,8,9]. Many ribozymes have been extensively characterized both in vitro and in vivo, yet it remains unclear how these RNAs acquired their catalytic capabilities. In this review, we first examine the difficulties in RNA evolution suggested by the results of experimental analyses, and then discuss the relationship between a stable three-dimensional structure and the evolution of alternative biochemical activities, using the bacterial *glmS* ribozyme as an example.

## 2. Fitness Landscapes of Ribozymes and Other RNAs

**Figure 1:**
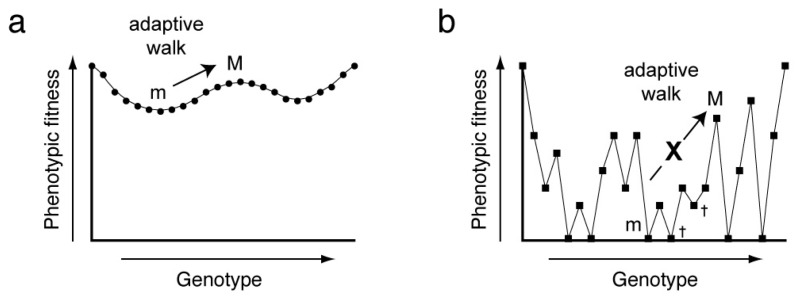
Fitness landscapes describe the relationship between the genotype and phenotype of an organism or sequence and the biochemical activity of a biopolymer. Fitness landscapes are very high-dimensional objects. For illustrative purposes, the genotype is reduced to a few dimensions. For this figure, the abscissa depicts a one-dimensional projection of all possible sequences (genetic distance) and the ordinate depicts the phenotypic fitness or biochemical activity. Evolution is an adaptive walk on the fitness landscape. (**a**) A smooth fitness landscape with many regions in which fitness varies monotonically with genetic change. A series of small or point mutations can lead without interruption from a local minimum (m) to a local maximum (M) of fitness; (**b**) A rugged fitness landscape, in which fitness varies unpredictably with genetic change. Adaptation is difficult for systems characterized by such surfaces, since many mutations will result in reduced fitness and mutants will be culled by natural selection (†) before a maximum can be achieved.

In the most general sense, the relationship between genotype and phenotype can be described by a fitness landscape, and adaptation is an optimizing walk on this high-dimensional surface. In its original formulation, the fitness landscape was envisioned as an *n* + 1-dimensional function relating all possible alleles of the *n* loci of a genotype to the phenotypic fitness [10]. The concept can be extended to describe the relationship between phenotype (e.g., biochemical activity) and all possible variants of a linear, genetically-encoded polymer (such as RNA or protein) of *n* residues [11]. Fitness landscapes can be smooth or rugged. In the former case, a series of small genotypic changes (point mutations) will allow continuous optimization of phenotypic fitness (Figure 1a). In the latter, point mutations will commonly be deleterious, and adaptation will be difficult, since most mutants will have reduced fitness and will be culled by natural selection (Figure 1b). Experimental construction of a fitness landscape for a biopolymer of more than a few residues is challenging as it requires both the synthesis of all possible sequence variants (*X^n^*, where *X* is the number of residue types, for instance 4 for RNA and 21 for proteins, and *n* is the length of the polymer), and the evaluation of the phenotypic fitness corresponding to each genotype. Advances in high-throughput sequencing methodology, combined with experimental designs in which the abundance of a sequence serves as a proxy for phenotypic fitness, have made it possible to experimentally examine the fitness landscapes of DNAs, RNAs and peptides in the past decade.

Even with the most efficient sequencing methods, the total sequence space of an RNA can only be examined exhaustively for short sequences [12]. The binding affinity of RNase P for its pre-tRNA substrates is modulated by the binding of the 6 nucleotide (nt) 5′ leader (which the ribozyme cuts off) to the C5 protein subunit of the ribozyme. Jankowski and coworkers synthesized a pre-tRNA pool containing all possible leader sequences and subjected it to cleavage by RNase P. By exhaustively sequencing the uncleaved population at multiple time-points, these authors evaluated the cleavage rate of each possible leader sequence, and from this, derived the affinity of each leader for the C5 protein [13]. This study provided a complete quantitative description of the protein-leader RNA interaction, and indicated that C5 does not bind to its physiological precursor tRNA sequences with the highest affinity, but rather, with an affinity that is closer to the mean of the distribution. The suboptimal affinity may reflect the requirement that the C5 subunit bind to many different tRNA leader sequences, allowing RNase P to process different tRNA precursors at comparable rates.

For longer RNA molecules, experimentally constructed fitness landscapes will necessarily be sparsely sampled. Pitt and Ferré-D’Amaré analyzed the fitness landscape of a previously in vitro selected 54-nt RNA ligase ribozyme by constructing a library containing 6 × 10^13^ sequence variants of the parental ribozyme, allowing it to self-ligate to a biotin-tagged substrate RNA. They used this tag to isolate reactive species at different time-points, and subjected these to high-throughput sequencing, thereby producing a sequencing-based apparent ligation rate for each sequence [14]. These authors separately synthesized all 162 possible point mutants of the ribozyme and biochemically determined their cleavage rates. Comparison of the sequencing-based and biochemical rates for all point mutants demonstrated a statistically robust correlation for sequence frequency enrichment and biochemical activity. By postulating that this correlation holds true for sequences that differ from the parental ribozyme by more than one mutation, the authors could construct a fitness landscape that describes how catalytic activity varies in sequence space. The resulting landscape was very rugged, with even very closely related genotypes exhibiting dramatically different phenotypic fitness. If true for other ribozymes, this would imply that catalytic RNAs are unlikely to maximize their activity through a smooth genotypic walk. A similar study recently performed on the twister ribozyme showed that of all the possible single and double mutants, 71% and 30%, respectively, retained at least 20% of the wild-type ribozyme activity. However, single and double mutations that affected the conserved structural elements of the catalytic RNA were severely deleterious [15]. Thus, while small genetic changes in proximity to the wild-type may be tolerated, this catalytic RNA is constrained in a local minimum by its structural requirements.

The ruggedness of RNA fitness landscapes is also reflected in the presence of multiple phenotypic peaks, which are not connected by genotypes that do not decrease fitness, i.e., the absence of neutral networks. Jiménez et al., through a combination of in vitro selection and high-throughput sequencing, generated a fitness landscape describing the ability of 24-nt RNAs to bind to guanosine triphosphate (GTP), finding a near-random distribution of disjoint fitness [16]. This study suggests that not only ribozymes but also aptamers evolve on rugged landscapes. If rugged landscapes are a characteristic of RNAs with activities that require the adoption of defined three-dimensional folds, genotypic random walks will mostly lead to evolutionary dead ends. So how may functional RNAs evolve? While there are many possible mechanisms, in the discussion that follows, some observations pertinent to compact, single-domain RNAs are considered.

## 3. Ribozyme Evolution through Intersection of Neutral Pathways

One possible solution to the conundrum posed by the rugged fitness landscapes inhabited by functional RNAs is that their evolution is not through optimization of one biochemical activity, but by the adoption of new capabilities. In a pioneering study, Schultes and Bartel examined such a scenario by constructing, in a step-wise manner, a mutational pathway that interconverted between an RNA ligase (the in vitro selected Class III ligase ribozyme) and an RNA cleaving ribozyme (derived from the hepatitis delta virus, HDV) [17]. Through manual analysis, these authors devised a sequence that lies 42 and 44 mutational steps away, respectively, from the ligase and the HDV ribozymes, and that may be able to adopt either of the unrelated folds. When this RNA was synthesized, it exhibited both catalytic activities, albeit at a very low level. However, a small number of mutations of this intersection making it more similar to either parental ribozyme produced variants that had more substantial biochemical activity. Therefore, in principle, evolutionary pathways exist that, with a finite number of mutations, would allow an RNA to adopt a new, unrelated fold and a new activity, maintaining the original activity until the new activity appeared.

Acquisition of a new catalytic function through stepwise mutation of an RNA may be challenging, however, even if the two chemistries are very similar. The pyrimidine nucleotide synthase ribozyme (^4S^U synthase), is a 124-nt in vitro selected ribozyme that catalyzes formation of the glycosidic bond between an activated ribose and 4-thiouracil [18,19]. Using a mutagenized RNA pool based on the ^4S^U synthase, an in vitro selection was performed to examine if closely related variants of the ^4S^U synthase that catalyze the synthesis of the purine nucleotide 6-thioguanosine (i.e., ^6S^G synthase) exist [20]. Despite the similarity between the two chemical reactions, the active ^6S^G synthase ribozymes that were isolated did not share any detectable structural similarity with the ^4S^U synthase. Structural probing and site-directed mutagenesis suggests that the ^6S^G synthases are structurally less complex than the ^4S^U synthases, being comprised of just two simple stem-loops. This work is consistent with the notion that, at least in some cases, evolution of new biochemical activities goes hand-in-hand with evolution of a new RNA structure.

## 4. Versatile and Promiscuous Ribozymes

Is a new structure necessary for each different ribozyme? Depending on how they are functionalized, many conserved and abundant protein folds (e.g., the triosephosphateisomerase or TIM barrel) are shared by enzymes that catalyze disparate reactions. Although the ligase-HDV and glycosidic bond synthase studies reviewed above may suggest that one RNA structure can only support one reaction, early ribozyme engineering studies using the Tetrahymena ribozyme, which is a considerably larger and more structurally complex RNA, argue otherwise. In addition to self-splicing, variants of this RNA have been reported to catalyze, to varying extents, a number of different reactions. Examples include RNA cleavage, RNA polymerization, and peptide bond hydrolysis [1,21,22,23]. More recently, pR1, a small promiscuous ribozyme, was described that can catalyze two different chemical transformations with comparable reaction rates [24]. pR1 was evolved by in vitro selection from a ^6S^G synthase, and can catalyze glycosidic bond formation between 6-thioguanine and either an activated or non-activated ribose (the former by nucleophilic displacement, the latter by Schiff-type chemistry), with comparable efficiency. This is a case of a ribozyme which, by using a promiscuous active site that can accommodate different substrates, can catalyze two reactions using the same overall architecture.

## 5. The *glmS* Ribozyme: One Fold, Two Catalytic Mechanisms

The *glmS* riboswitch-ribozyme [25] is unique among natural ribozymes in that it employs an exogenous small molecule as a co-enzyme for catalysis [26]. In its biological context in Gram-positive bacteria, this ligand-dependence is harnessed for the regulation of gene expression. The coenzyme of this ribozyme is glucosamine-6-phosphate (GlcN6P), an essential precursor for cell wall biosynthesis. Binding to GlcN6P activates self-cleavage by the ribozyme, leading to degradation of the RNA [27]. As the ribozyme is part of the mRNA encoding the protein enzyme responsible for GlcN6P synthesis, this results in negative feedback control. This biological role makes this ribozyme a riboswitch [28] as well (albeit an atypical one, as most riboswitches function by transcriptional or translational control).

The *glmS* ribozyme is a relatively efficient catalyst, attaining a maximal cleavage rate of ~100 min^−1^ in the presence of GlcN6P, comparable to the rates of some of the fastest known ribozymes, such as the Class I ligase (maximum rate of 800 min^−1^) [29]. Consistent with the abundance of GlcN6P in bacteria, this RNA has a modest affinity for its coenzyme (*K*_d_ ~ 300 mM) [30], and exhibits only limited chemical specificity. Various analogues of GlcN6P, such as glucosamine, l-serine and Tris (tris(hydroxymethyl)aminomethane), which all share vicinal amine and hydroxyl groups in equivalent stereochemical relationships, can activate the ribozyme to various extents (d-serine is not an activator) [31]. In principle, GlcN6P could function as either an allosteric activator or a coenzyme that provides a catalytically critical functional group to the active site of the ribozyme.

**Figure 2:**
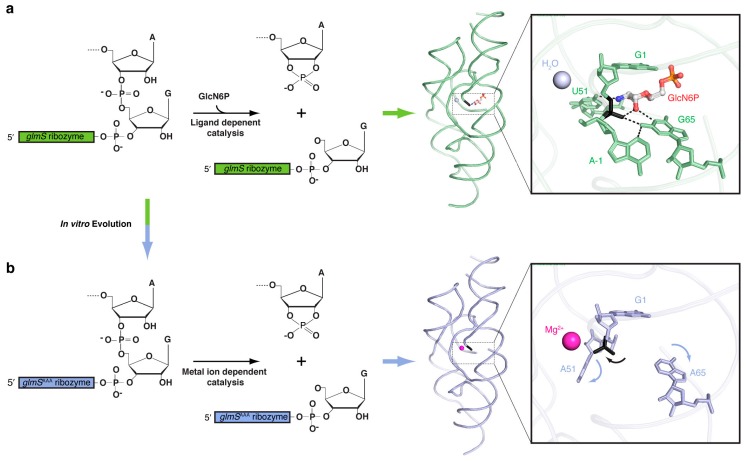
In vitro evolution of the co-enzyme independent, triple adenosine-mutant *glmS* metalloribozyme (*glmS*^AAA^) from the GlcN6P-dependent wild-type ribozyme (*glmS*^WT^) [37]. Global and active site structure of (**a**) *glmS*^WT^ in green and (**b**) *glmS*^AAA^ in light purple. Scissile phosphate is colored in black, and hydrogen bonding interactions are shown in dotted lines. Arrows depict the displacement of nucleobases (light purple) and the scissile phosphate (black) in the *glmS*^AAA^ structure as compared to *glmS*^WT^.

The crystal structure of the *glmS* ribozyme provided the first glimpse of the catalytic mechanism of this ~145-nt RNA [32,33]. The RNA adopts a complex architecture stabilized by three pseudoknots. The active site, which is defined by the position of the scissile phosphate, is immediately adjacent to the GlcN6P binding site. X-ray crystallographic analysis revealed that the ribozyme binds GlcN6P in such a manner that the amine of the sugar is in van der Waals contact with the 5′-oxo leaving group of the internal transesterification reaction (Figure 2a). This immediately suggests that the GlcN6P could be functioning as a general acid catalyst. Indeed, glucose-6-phosphate, which differs from GlcN6P only in lacking the amine group, is a competitive inhibitor [31], and the apparent pKa (acid dissociation constant) of the ribozyme reaction tracks the p*K*_a_ of the amine group of GlcN6P and other non-physiological activators [31,34]. Biochemical and structural studies demonstrated that the *glmS* ribozyme does not change conformation upon GlcN6P binding, cleavage, or release of GlcN6P post-reaction [32,35,36]. Thus, GlcN6P is not an allosteric activator, and functions solely as an obligate coenzyme. The ribozyme is completely inactive in the absence of GlcN6P, even with the cleavable RNA strand bound, demonstrating that its active site has evolved to rely on the exogenous GlcN6P for activity.

The *glmS* ribozyme is, thus far, the only known natural ribozyme that has an absolute requirement for an exogenous coenzyme. Since many other ribozymes that catalyze the same sequence-specific RNA cleavage reaction through internal transesterification (using either RNA functional groups or bound cations) are known, the discovery of the general acid catalyst function for GlcN6P in the *glmS* ribozyme raised a fundamental question about its evolutionary origin. Did the *glmS* ribozyme evolve from a GlcN6P-binding RNA (e.g., a conventional riboswitch) by becoming catalytic, or did it evolve from a coenzyme-independent ribozyme that became dependent on GlcN6P? Because of the exceptional structural stability of the *glmS* ribozyme fold, experiments addressing this question would also illuminate the broader question of whether a stable RNA fold can be decorated with different residues to support multiple biochemical activities.

These questions were addressed through an in vitro evolution study of the *glmS* ribozyme [37]. In this work, we first generated an RNA pool containing 7.5 × 10^14^ *glmS* ribozyme variants in which residues in proximity to the binding site were mutagenized, without affecting any of the base pairing interactions that define the secondary structure or the three pseudoknots that confer on the RNA its exceptional stability. We then challenged the RNA sequences to catalyze internal RNA cleavage in the absence of GlcN6P, and only those sequences that were active were iteratively amplified and enriched. The selection resulted in the isolation of a *glmS* ribozyme variant bearing three adenosine mutations (*glmS*^AAA^) that can catalyze the same site-specific internal transesterification reaction as the wild-type in the absence of GlcN6P. This mutant can cleave RNA at rates of up to 2.5 min^−1^ in the complete absence of GlcN6P, requiring only divalent cations for activity (and its activity is not enhanced by GlcN6P). Crystallographic and small-angle X-ray scattering (SAXS) analyses demonstrated that *glmS*^AAA^ retains the overall fold of the wild-type ribozyme (Figure 2). A combination of crystallographic and phosphorothioate substitution experiments suggested that *glmS*^AAA^ employs a divalent cation to neutralize the anionic leaving group of the cleavage reaction, being therefore a metalloenzyme (Figure 2b).

To analyze the possible evolutionary pathway between a GlcN6P-independent ancestral ribozyme, such as *glmS*^AAA^, and the contemporary ribozyme, we constructed all the single and double revertants of the three mutations present in the former. Remarkably, point reversions were sufficient to confer GlcN6P-dependence to the catalytic RNAs, and additional reversions gradually suppressed all coenzyme-independent cleavage. Thus, a neutral pathway that is as short as possible (a single point mutation) connects the metalloribozyme *glmS*^AAA^ with the strictly dependent wild-type *glmS* ribozyme. Moreover, at least for this highly stable RNA fold, one overall architecture can support two different catalytic activities. This suggests that despite the rugged fitness landscapes that appear to characterize ribozymes, because different biochemical activities can arise through minimal (i.e., as few as one) mutations, these catalysts may evolve by the rapid acquisition of new biochemical functions [37].

## 6. RNAs and Ribozymes in Bioengineering

Advances in the study of RNA structure and function in the past decade have not only increased knowledge of RNA biology, but also encouraged the engineering of RNAs as biotechnological tools. The characterization of cellular components involved in small RNAs regulatory pathways, for example, have led to the development of silencing RNA (siRNA) therapeutics for targeting specific cancer and disease related genes [38]. The clustered regularly interspaced short palindromic repeats (CRISPR) bacterial-phage defense mechanism [39] is another example of a biological system that was successfully developed into a technology that is now widely used for site-specific genome editing and in epigenetic studies.

Riboswitches and ribozymes have also been expanded into synthetic biology and engineered to perform complex functions in cells that have also been explored as possible players in the development of RNA therapeutics. The Breaker group, for example, engineered an allosteric ribozyme by fusing an ATP-binding RNA motif (aptamer) with the self-cleaving hammerhead ribozyme. Binding of either adenosine or ATP drastically reduces the ribozyme activity, demonstrating how RNAs can be subjected to allosteric regulation using an exogenous small molecule. Using a similar RNA fusion approach, the Breaker lab, along with other research groups, have since engineered synthetic RNAs using the aptamer region as a genetic switch to control the expression of specific proteins [40]. These RNAs, known as the “aptazymes”, undergo a ligand induced structural rearrangement that folds the ribozyme into an active conformation. Activation of the ribozyme results in a self-cleavage reaction in *cis*, which leads to the degradation of the mRNA and silences the expression of the downstream genes.

In vitro evolution of ribozymes with pre-existing functions, as in the studies of the *glmS*^AAA^ ribozyme described above [37], offers an alternative approach for the discovery of RNAs with unique functions. By retaining a stable RNA structural scaffold, we have demonstrated that it is possible to convert the *glmS* ribozyme with strict dependence on GlcN6P to a metalloribozyme requiring only divalent metal ions for catalysis. Further ribozyme engineering by in vitro selection or mutational studies based on known RNA structures can therefore be a fruitful approach to rationally design and engineer new ribozymes with novel biological activities.
